# Effects of Forest Age and Invasive Shrubs on Mycophilous Coleoptera Communities in a Temperate Deciduous Woodland

**DOI:** 10.3390/insects16070735

**Published:** 2025-07-18

**Authors:** Jeffrey M. Brown, John O. Stireman

**Affiliations:** Department of Biological Sciences, Wright State University, Dayton, OH 45435, USA; jefjefjef@hotmail.com

**Keywords:** old-growth forest, detritivore community, mycophagy, beetles, diversity, invasive plants, forest fungi

## Abstract

Forests in the Eastern and Midwestern U.S. have been profoundly affected by human use over the last 150 years, with few old growth forests remaining. Such mature forests may harbor distinct communities and high biodiversity, particularly detritivores and their associated food webs. These communities, however, have been surveyed only rarely in comparisons of diversity and community composition between old and young forests. Here, we compare the mycophilous beetle communities of young and old deciduous forest stands in Southwestern Ohio (U.S.A.). We assess how the abundance and diversity of beetles associated with fungal sporocarps varies with forest age, downed woody debris, and invasive honeysuckle density. We surveyed fungus-associated beetles with baited traps at eight wooded parklands centered around Dayton, Ohio, conducting sampling three times over a growing season. In contrast to expectation, we found no clear effect of forest age on mycophilous beetle communities, but infestation by invasive honeysuckle (*Lonicera maackii*) negatively affected beetle abundance and diversity. Beetle abundance, richness, and community composition also strongly varied across seasonal sampling periods. Our surveys of mycophilous beetles in a Midwestern U.S. forest represent an initial step toward understanding how these communities are shaped by forest age and invasive species. Such information is crucial in managing forests to preserve biodiversity and ecosystem services.

## Simple Summary

Mature, old growth forests are known to harbor distinct plant and animal communities and high biodiversity. However, there has been little study of how detritivore communities vary in diversity and composition between old and young forest stands. To assess how such communities vary with forest age, we compare fungus-associated beetle communities in young and old deciduous woodlands of Southwestern Ohio (U.S.A.) over a growing season. We found no strong differences in abundance or diversity of fungus-associated beetle communities between new and old forest stands, but infestation by invasive honeysuckle shrubs was associated with decreased beetle abundance and diversity. In addition, communities strongly varied across seasonal sampling periods. Our surveys of these fungus-associated beetles represent an initial step toward understanding deciduous forest detritivore communities in the U.S. Midwest and how they are shaped by forest traits. Such information is crucial if we wish to manage forests to enhance biodiversity and ecosystem services.

## 1. Introduction

Much of the original forest in the eastern Midwest U.S. has been lost or substantially degraded over the past 150 years. For example, in Ohio, it has been estimated that forest cover approached 95% when the first European settlers arrived. However, through logging and land clearing for agriculture, it was reduced to as low as 10% in the early 1900s and is even lower in glaciated areas of western Ohio. The impact of this extensive land use change on forest biodiversity is difficult to estimate; however, it is clear that tracts of mature forest, which may harbor high levels of unique biodiversity (e.g., [1,2,3]), have been greatly diminished across much of the region [3].

A number of studies have surveyed the biodiversity of various groups of organisms, including understory plants [4,5], birds [6], moths [7], and fungi [8], in remnant mature temperate forests of Ohio and other regions. Such studies have often found old growth forests to harbor distinct and sometimes more diverse communities relative to second growth forests (e.g., [4,9]). An important component of biodiversity and ecosystem processes that is not often included in biodiversity surveys is the saprophage/detritivore community. Detrital foods webs are essential to the overall health of ecosystems through nutrient cycling, returning inaccessible nitrogen, phosphorous, carbon, and other minerals to an available state [10]. Furthermore, communities of detritivores, fungivores, and xylophages are expected to be less abundant and diverse in young forest stands than in older, more mature forest remnants [2]. In the current study, we focus on one component of the detritivore community, mycophilous beetles. We briefly review the associations between beetles and fungi, assess how mycophilous beetle communities vary between young and older forest stands, and examine how these communities are affected by plant invasion and resource availability (coarse woody debris).

Fungi, and the organisms that feed on them, are integral components of forest detrital food webs. Fungi are heterotrophic, relying on other organisms for their nourishment. The fruiting bodies or sporocarps of fungi are visible as mushrooms and shelf fungi with the bulk of the organism living inside their substrate as hyphae. Fungi fill key roles in forest ecosystems by forming nutritive and protective symbiotic relationships with plants [11,12]; acting as saprotrophs and decomposing plant matter, particularly the recalcitrant compounds cellulose and lignin [13]; and functioning as obligate or facultative parasites of plants and animals. The sporocarps of saprotrophic fungi, which often rely on dead woody debris, provide an important food resource utilized by many forest animals, both vertebrates and invertebrates. In forests, fungal diversity is thought to be positively correlated with vascular plant diversity [14], but there may be other factors, such as disturbance and plant invasion, forest age [15], moisture [16], amount of downed woody debris, and soil temperature or soil pH [17], that determine fungal abundance and diversity.

Among the most diverse and important groups of animals dependent on forest fungi are the Coleoptera (beetles), which include a large number of species across many families that are associated with fungi. Associations between beetles and fungi have a long evolutionary history, and mycophagy has arisen many times across disparate lineages within the order [18]. Fungal sporocarps attract some beetles that use them as a food resource, as well as others that seek a source of prey [19]. As most fungal sporocarps are an ephemeral and unpredictable resource, beetles that use them as a resource have evolved well-developed dispersal abilities and rapid life cycles [20]. Some Coleoptera form obligate symbiotic relationships with fungi, such as beetles in the subfamily Scolytinae with ambrosia fungi [21]. Other relationships may be more one-sided with beetles simply using the fungi as a food source, but even then, there is some evidence that feeding behaviors assist in spore dispersal [22,23]. Beetles associated with ephemeral fungal sporocarps tend to be generalists, while those associated with persistent sporocarps such as bracket fungi may be more specialized [24]. Some mycophagous beetle groups, such as members of the staphylinid genus *Oxyporus*, exhibit a wide range of host specificity, ranging from generalist (utilizing fungi belonging to eight families) to exclusively using a single fungal species for their life cycle [25]. Mycophilous beetles may serve as indicator species of forest health and ecosystem function because the diversity and abundance of mycophilous beetle communities are dependent on the diversity and abundance of the fungal community (e.g., [26]), which itself is dependent on environmental variables such as available substrate (e.g., downed woody debris and tree diversity), abiotic conditions such as moisture and humidity, and disturbance [27,28,29].

Several studies have sought to characterize the relationships between beetles and fungi in various forest ecosystems. For example, Klimaszewski and Peck [30] found that there is a succession of beetle communities that turn over as *Cerioporus* (aka *Polyporellus*) *squamosus* sporocarps age. Cline and Leschen [31] created a checklist of beetles found on the common oyster mushroom, *Pleurotus ostreatus*. Several studies have also examined beetle communities associated with fungi in coniferous forests of Europe [32,33]. One European study [34] found that there was a moderate positive relationship between deadwood volume and species richness of saproxylic beetles, which was stronger in boreal than temperate forests. In one of the few studies of fungus-associated beetle communities in Eastern U.S. forests, Epps and Arnold [26,35] found that beetle abundance was positively related to the mass of particular sporocarps and that beetle diversity increased with sporocarp age.

Despite insights gained from studies of mycophilous beetle communities, we know very little about how these communities vary across habitats or in relation to environmental factors. In particular, the relationship between mycophilous beetle communities and forest age (mature versus young second growth) has not been previously explored in temperate deciduous forests. Due to elevated shade, moisture, and downed woody debris, it may be expected that mycophilous beetle abundance and diversity is greater in older forest stands and that these stands might harbor distinct mycophilous beetle assemblages. Furthermore, young forest stands may be more likely to be invaded by nonnative plant species, which could influence mycophilous beetle communities through effects on their fungal hosts [36].

The primary aim of the current study was to assess how forest age influences the abundance and diversity of mycophilous beetles. In addition, we examine the effects of resource abundance (course woody debris) and extent of woody plant invasion in these communities. In exploring this issue, we provide an initial characterization of the community of mycophilous beetles associated with a deciduous forest in the Midwestern U.S. To our knowledge this important ecological guild has not been studied in this region.

## 2. Materials and Methods

### 2.1. Study Area

The remaining woodlands of the Miami Valley region of Southwest Ohio are primarily of the oak–hickory (*Quercus–Carya*) and beech–maple (*Fagus*–*Acer*) forest types, with an abundance of elm (*Ulmus*) and ashes (*Fraxinus*; although the invasive emerald ash borer, *Agrilus planipennis* Fairmaire [Buprestidae], has killed most mature ash trees in the region). Additional common tree genera in the region include *Prunus* (cherry), *Juglans* (walnut), and *Celtis* (hackberry). These woodlands are in various stages of succession, with very few stands of old growth forest remaining. The more mature forest stands often have a greater abundance of dead woody debris, as well as fallen leaves and other decaying organic matter, which is expected to promote the abundance and diversity of saprotrophic fungi. Forests of the region are heavily invaded by Amur honeysuckle (*Lonicera maackii*). Although this large woody shrub is most dominant along forest edges and in younger successional forests, it is shade tolerant and able to invade the interior of mature forests as well. This species has been shown to significantly impact plant communities and decomposition dynamics [37,38,39].

### 2.2. Beetle Surveys

**Figure 1:**
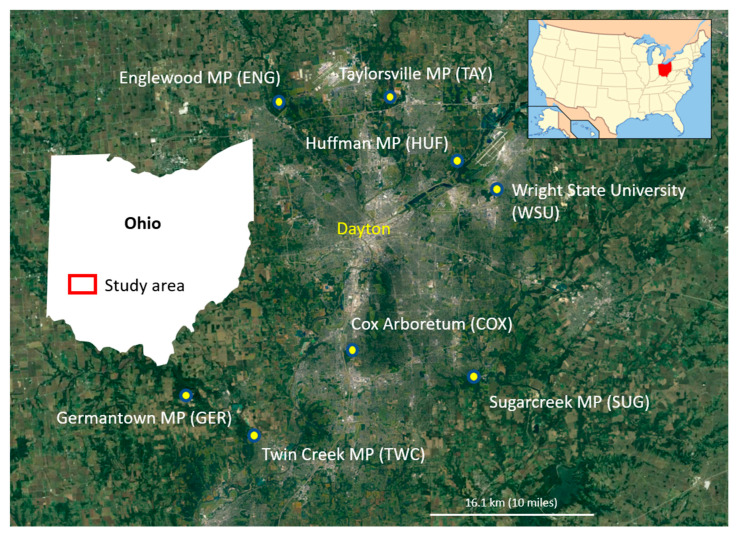
Study sites in the Miami Valley region of Southwest Ohio with site abbreviations. The upper left inset map shows the location of Ohio within the U.S.A. (red), and the left inset map shows the location of the study area within Ohio (red rectangle).

We surveyed fungus-associated beetles in old and young forest stands at eight sites surrounding the greater Dayton area in Southwestern Ohio (Figure 1). These eight sites included the Wright State University woods (WSU), as well as Englewood (ENG), Huffman (HUF), Taylorsville (TAY), Germantown (GER), Twin Creek (TWC), Cox Arboretum (COX), and Sugarcreek (SUG) Metroparks (Figure 1, Table S1). Each site was selected to be a protected natural area with an area of contiguous forest of at least 50 hectares, containing stands of both older mature forest and younger successional forest growth in close proximity.

To select sampling sites, we examined historical aerial photographs from the Greene (1940) and Montgomery County (1950–1960) archives. We selected old forest sites that had well established forest cover in both historical photographs and recent (2018) Google Maps satellite views. Considering the latest aerial photo year (1960), the mature forests were a minimum of 80–100 years old and probably much older as they were already clearly established. We selected young forest sites that had little to no forest cover in the historical photographs but did have forest cover in recent Google Maps satellite views. We visited each of these sites before setting up beetle traps to verify that the site matched the satellite views (i.e., there had been no recent clearing) and that we could establish a 40 m transect that was at least 10 m from any trail or edge habitat. We attempted to select similar, relatively level sampling locations across all sites. We avoided locating sampling locations in areas with obviously human-planted trees or with large numbers of conifers, as the study is focused on native deciduous forests.

**Figure 2:**
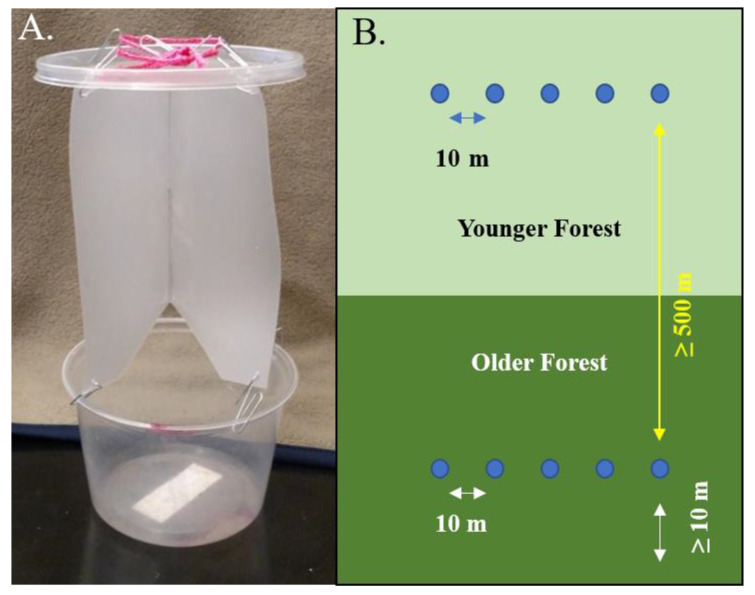
(**A**) Simple cross vane flight intercept trap used to sample beetles. (**B**) Schematic diagram of the placement of flight intercept traps (blue dots) at each sampling site.

We constructed small cross vane flight intercept traps (estimated catch area ca. 616 cm^2^; Figure 2A), which were suspended 1 m above the forest floor to minimize disturbance from small mammals. As darker colored traps resemble trees, we used a lighter color to mimic fungi and to reduce bycatch. The traps simulated fungal resources as they were baited with the Oyster mushrooms, *Pleurotus ostreatus*, a species known to attract a wide variety of mycophagous Coleoptera [31]. All mushrooms were purchased at the same grocery store the day the traps were placed. Approximately 10 g of bait was wrapped in cheesecloth and attached to a randomly chosen trap with a small binder clip. Non-toxic antifreeze (propylene glycol) was used as a killing agent and preservative in the collecting basin of each trap. We used suspended traps rather than pitfall traps, because in preliminary sampling using baited pitfall traps, 90% of traps were disturbed by wild mammals.

At each site, we hung five flight intercept traps in both older and younger forest stands, for a total of 10 traps per site (80 traps total). The traps were placed in a straight line, 10 m apart, as much as terrain allowed (Figure 2B) and a minimum of 10 m from the edge of the forest to reduce possible edge effects (the only site in which traps were less than 30 m from the forest edge was the Huffman MP young woods plot, in which the closest trap was ca. 15 m from the forest edge). The old and young sampling locations at Cox Arboretum were the closest in proximity (ca. 500 m apart); however, in the other parks, the distance between paired sampling locations was at least 1 km. This distance was chosen to maximize impendence of samples in young and old forest plots while still sampling in the same forested area and general community.

As fungal sporocarp production is known to vary seasonally [40], we sampled each site three times, once in the early summer season (week beginning 4 June 2018, “early summer”), once mid-summer (week beginning 23 July 2018, “mid-summer”), and once late season (week beginning 1 October 2018, “fall”). The same trap locations were used in each successive sampling period. Each trap ran for two days, after which we collected all specimens and stored them in sealed bags with a small amount of propylene glycol.

For the fall sampling period, the first three trap locations at Huffman Metropark were destroyed. The trees were cleared by the Miami Conservancy District in area around a water monitoring well. We replaced those three traps in a line continuing past the two remaining undisturbed trap locations.

Once back in the lab, samples were stored in a freezer until they were processed. We extracted all Coleoptera from the trap samples and transferred them to vials of 70% ethyl alcohol. We did not examine the remaining insect material, which mostly consisted of Diptera and Hymenoptera. At least one specimen of each morphospecies was pinned or pointed to aid in identification, depending on the size of the specimen. If we were not sure a specimen matched a previously pinned morphospecies, we pinned it for later comparison.

Beetle specimens were keyed out at least to family using *American Beetles* volumes 1 and 2 [41]. As some taxonomy has changed since these texts were published, we updated names where available. Most specimens were keyed to genus as well using [41], and some were identified to species using the available literature. Many of the genera lack current dichotomous keys. All specimens were assigned to a described species or morphospecies based upon careful microscopic examination of external morphological features. We were conservative in assigning morphospecies, lumping specimens together if their external morphology, color, and size did not present obvious differences. True species richness is likely much higher, particularly as some of the very small beetles (<2 mm) required specialized identification techniques (e.g., genitalic dissection) outside the scope of this study.

We further narrowed the number of specimens by restricting focus to members of families with known fungal associations according to Arnett & Thomas [41] and Evans [42]. We include as “mycophilous” not just taxa that specifically feed on fungi as adults or larvae but also predators of fungus-associated organisms or that have other potential associations with fungi. Given our incomplete identifications and the limited natural history information available for many genera and species, we adopted this objective criterion as a compromise between excluding potentially mycophilous species and including species with no fungal association; however, we acknowledge that it may include some incidental taxa. According to this criterion, 97.6% of trapped beetles belonging to 35 families with known fungal associations were retained. Although this overestimates which individual species were associated with fungi, it suggests that there was a relatively small proportion of bycatch, validating our collection method. All further results and analyses are restricted to this subset of 2873 beetles from fungal associated families. Beetle morphospecies vouchers are deposited in the collection of John Stireman at Wright State University (JOSC) and are available for loan or study.

Using a modified point-centered quarter method [43], we selected up to eight trees with a minimum diameter at breast height (DBH) of 10 cm measured 1.5 m from the forest floor (the two trees closest to each trap per quarter) and recorded the tree species and DBH (see Table S1 for included species). We converted the DBH from cm to inches and multiplied by the estimated tree growth factor (Table S2) from Purdue University [44] to obtain the estimated tree age. Any tree growth factors not available were estimated using values for related species and tree growth rates from the Morton Arboretum [45]. We then calculated the average age (using all measured trees) and the average maximum age (using the oldest measured tree per trap) of trees at each site.

We walked a 50 m transect along each line of traps to quantify the amount of coarse woody debris (CWD). We measured the diameter at each end of all sticks and logs that crossed this transect that were at least 5 cm in diameter. We stopped measuring at 5 m to either side of transect if the CWD continued and stopped measuring if the diameter dropped below 5 cm. We estimated the volume by averaging the area of the two ends and multiplying by the length. We then assigned a decay class of 1–5 according to Angers et al. [46]. In order to combine the five decay classes into one variable for analysis, we weighted each class by their number and summed the results to give a CWD score. The scores were weighted as later stages of wood decay have increased abundance and diversity of fungi [47], potentially attracting a greater abundance and diversity of Coleoptera.

We quantified the basal area of invasive Amur honeysuckle (HS), *Lonicera maackii*, by measuring the basal stem area of all plants at least 1 cm in diameter in one 5 m × 5 m quarter of each trap and then summed these values across the five traps at each sampling location.

### 2.3. Statistical Analysis

Once all morphospecies were assigned, we created an overall rarefaction curve with *iNEXT* [48] to determine how well our sampling efforts reflected the total estimated number of species. We extrapolated values of the rarefaction curve to determine the approximate sampling effort needed to reach the total estimated species. We also examined rarefaction curves for old and young woods and sampling period separately.

We used linear mixed effect models with the *lme4* package [49] to analyze the effects of environmental variables on beetle abundance and richness. Sampling site (park) was assigned as a random effect in all models, as each site likely had other environmental factors that were not recorded which could affect the intercept. Preliminary analysis indicated no relationship between forest stand size and beetle richness or abundance; thus, forest area was excluded from subsequent models.

In models with abundance as the response variable, quantile–quantile (Q–Q) plots revealed skewed distributions, and a Shapiro–Wilk normality test indicated that abundance was not normally distributed (*p* < 0.001). We applied a Log_2_ transformation to achieve a more normal distribution (*p* = 0.201). We selected the minimal best model with forest age class (old/young), sampling period, and HS as predictors based on chi-square comparisons of several models, including interactions. This minimal best model was a better fit according to AIC than the full model (ΔAIC = 1.51). A model with only sampling period and HS performed marginally better (ΔAIC = 0.4) but did not include forest age class, our primary variable of interest. We assessed if honeysuckle (HS) infestation levels varied significantly among mature and young woods with a nested ANOVA (stand age within sampling site).

For species richness, Q–Q plots showed no obvious departures from normality (Shapiro–Wilk *p* = 0.385), and we selected a simplified model with age, sampling period, and HS as predictors based on an ANOVA comparisons of several models, including interactions. This model was a marginally better fit according to AIC than the full model (ΔAIC = 0.16). We also estimated simple linear models to compare the relationship of species richness to total abundance, as well as Shannon effective species (*e*^H^) to total abundance.

In order to visualize clustering among sample sites and dates with regard to beetle community composition, we created nonmetric multidimensional scaling (NMDS) plots of the entire data set of fungus-associated beetles using the metaMDS function from the vegan package [50] in R v.2.6-4. The input was a site by species abundance matrix of the 48 possible site/age/sampling period combinations and the 211 recognized morphospecies standardized to relative abundances by dividing by marginal totals. We used the Bray–Curtis dissimilarity and a k value of 4, which provided a reasonable stress value (0.083) and a high non-metric R^2^ (0.993). Effects of site, forest age, and sampling period on community composition were assessed using analysis of similarity (ANOSIM) in vegan.

To examine similarity of beetle species occurrence between sites and old versus young woods, we calculated Jaccard Indices. We converted the data to binary species presence/absence and the used the vegdist function from vegan to compute dissimilarity measures, which we then converted to similarity as they are more intuitively understood. We computed the means of Jaccard similarity scores to examine if sampling locations were more similar within a park or across age categories.

## 3. Results

### 3.1. Beetle Abundance and Richness

In total, the traps collected 2943 beetles of 35 families from 240 trap samples, with 2873 being potentially fungus associated. The traps varied greatly in the number of beetles recovered. Nine (4%) of the trap samples were disturbed by wildlife, likely deer or squirrels, and had no beetles. The number of beetles within individual undisturbed traps ranged from zero (three traps from the third sampling period) to 70 (one trap from the early summer sampling period), with a mean of 12.74 beetles per trap.

**Figure 3:**
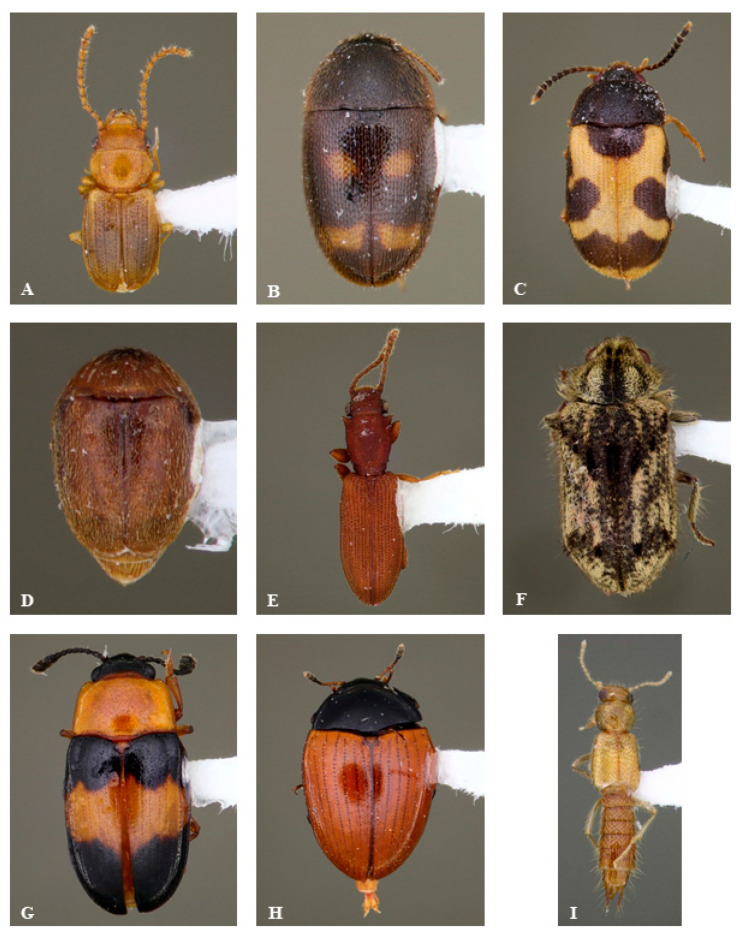

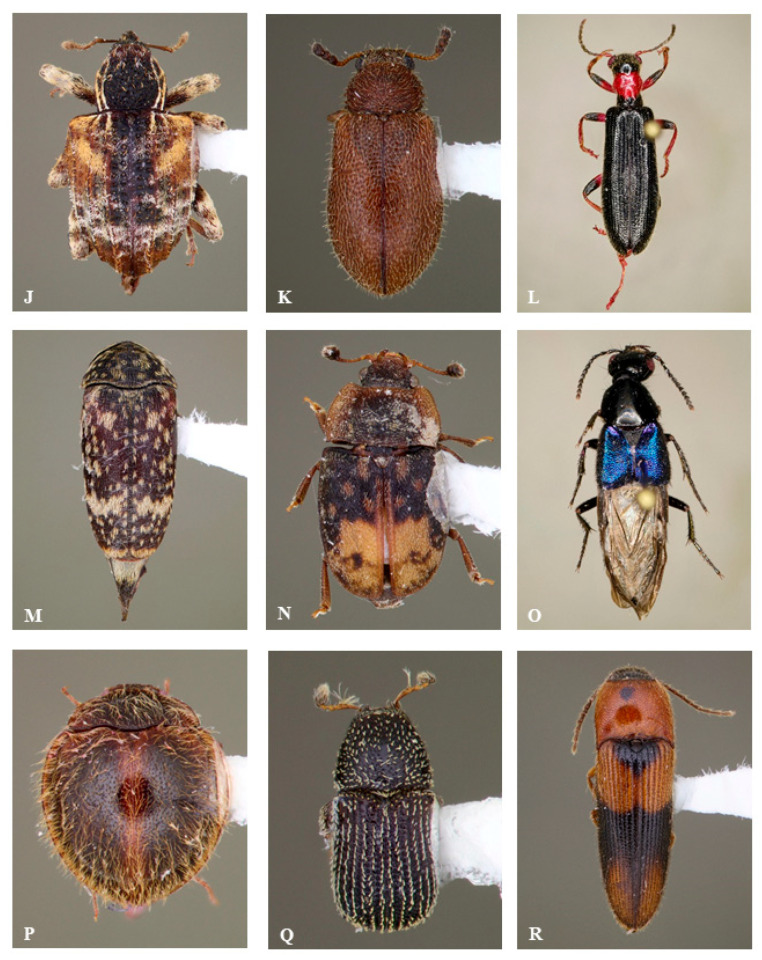
Images of representative beetle species collected in traps: (**A**) Laemophloidae: *Charaphloeus* sp. (**B**) Mycetophagidae: *Litargus tetraspilotus* LeConte. (**C**) Mycetophagidae: *Mycetophagus punctatus* Say. (**D**) Corylophidae: *Sericoderus lateralis* (Gyllenhal). (**E**) Silvanidae: *Silvanus muticus* Sharp. (**F**) Ptinidae: *Trichodesma klagesi* Fall. (**G**) Erotylidae: *Triplax festiva* Lacordaire. (**H**) Erotylidae: *Tritoma sanguinipennis* (Say). (**I**) Staphylinidae: *Palaminus* sp. (**J**) Curculionidae: *Conotrachelus anaglypticus* (Say). (**K**) Latridiidae: *Corticaria* sp. (**L**) Cleridae: *Cymatodera bicolor* (Say). (**M**) Mordellidae: *Mordellaria serval* (Say). (**N**) Nitidulidae: *Omosita nearctica* Kirejtshuk. (**O**) Staphylinidae: *Philonthus caeruleipennis* (Mannerheim). (**P**) Coccinelidae: *Scymus* sp. (**Q**) Curculionidae: *Phloeotribus* sp. (**R**) Elateridae: *Ampedus areolatus* (Say).

**Figure 4:**
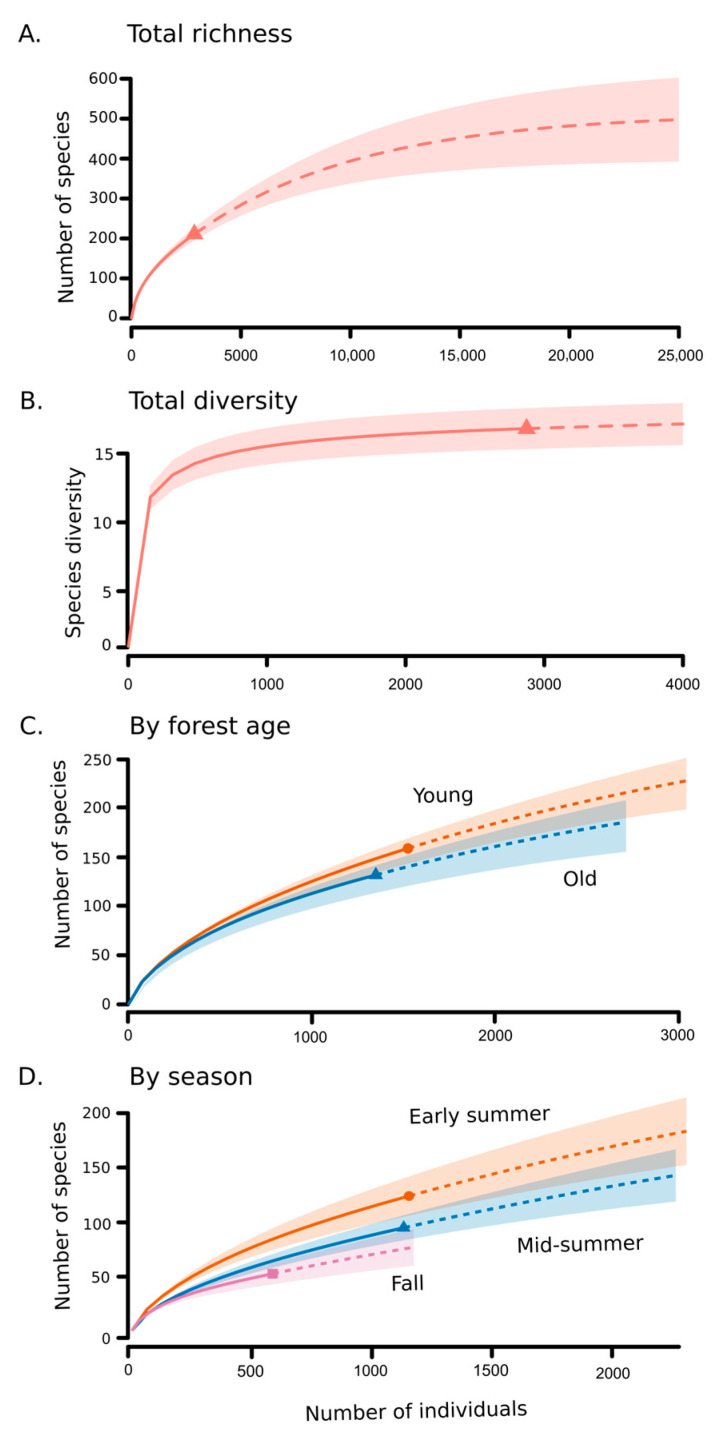
Species accumulation curves for total beetle species (**A**) and total effective species (**B**) by forest age (**C**) and by sampling period (**D**). Shapes indicate totals observed, solid lines indicate rarefaction curves, and colored regions indicate approximate 95% confidence intervals. Dotted lines are extrapolations to approximate saturation in (**A**,**B**) and to double the sample size in (**C**,**D**). Note: Scale of x and y axes varies among panels.

We were able to recognize 211 beetle morphospecies (Figure 3, Table 1 and Table S3) from the trap samples; however, this likely represents an underestimate of the species richness present, particularly for small taxa. The total extrapolated number of beetle species present in the community based on rarefaction was estimated to be 514, of which our samples captured 41% (Figure 4A). In order to sample the entire 514 estimated species expected to occur, a much higher sampling effort of approximately 25,000 specimens would be required. This estimate is restricted to beetles captured by this method, as suspended flight intercept traps may not capture all beetle taxa.

Overall, our sampling collected 92% (16.8 observed/18.3 estimated) of the Shannon effective species (*e*^H^, Figure 4B). Although we found more total species across sites in the young woods (Figure 4C), the older woods sites had a higher number of effective species, suggesting that the old woods sites had higher evenness as indicated by the Simpson diversity index (Table S4).

**Figure 5:**
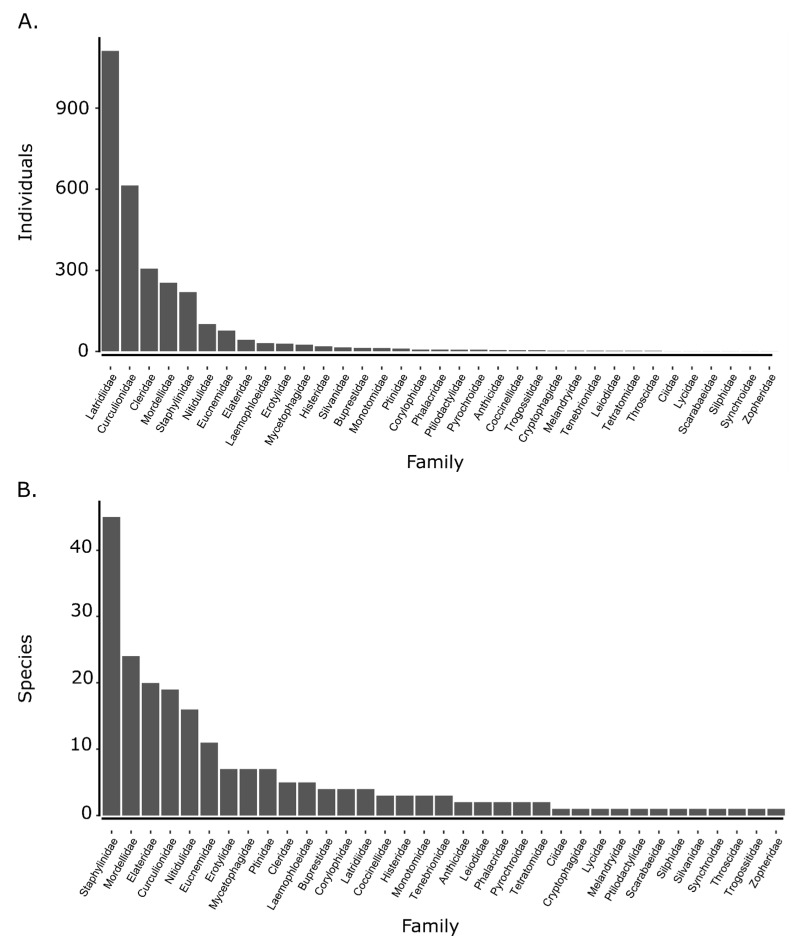
Total abundance (**A**) and richness (**B**) by beetle family, ordered from highest to lowest, across all traps and sampling periods.

The five dominant beetle families by abundance (Latridiidae: 1112, Curculionidae: 614, Cleridae: 306, Mordellidae: 254, Staphylinidae: 219) comprised 85% of individuals collected, with Latridiidae alone composing 38% (Figure 5A). Family rankings for species richness were quite different than for abundance, and the distribution of species richness was somewhat more even across families. The five dominant families by species richness (Staphylinidae: 45, Mordellidae: 24, Elateridae: 20, Curculionidae: 19, Nitidulidae: 16) comprised 58% of species collected, and Staphylinidae alone comprised 21% (Figure 5B).

### 3.2. Effects of Forest Traits

Comparing our site designations of old and young woods to estimated tree ages (Table 2) generally provided support for our age classification, as only one site (ENG) did not match our classification when comparing the average maximum tree age, and two sites (ENG and SUG) departed from our classification in terms of average tree age. The young woods Englewood sampling location had several large relic Osage orange (*Maclura pomifera*) trees that skewed the average age estimates (see Table S1 for measured species).

We found no consistent pattern of volume and decay classes of coarse woody debris between old and young woods within sites or between old and young woods across all sites (Figure S1). Decay classes 3 and 4 were the most common, together comprising nearly 80% of CWD volume, with decay class 1 (freshly fallen) being scarce and only representing 0.7% of CWD volume. The Sugarcreek Metropark young site had a high volume of downed wood, which appeared to be ash (*Fraxinus* spp.) killed by emerald ash borer (*Agrilus planipennis*) as evidenced by larval galleries.

**Figure 6:**
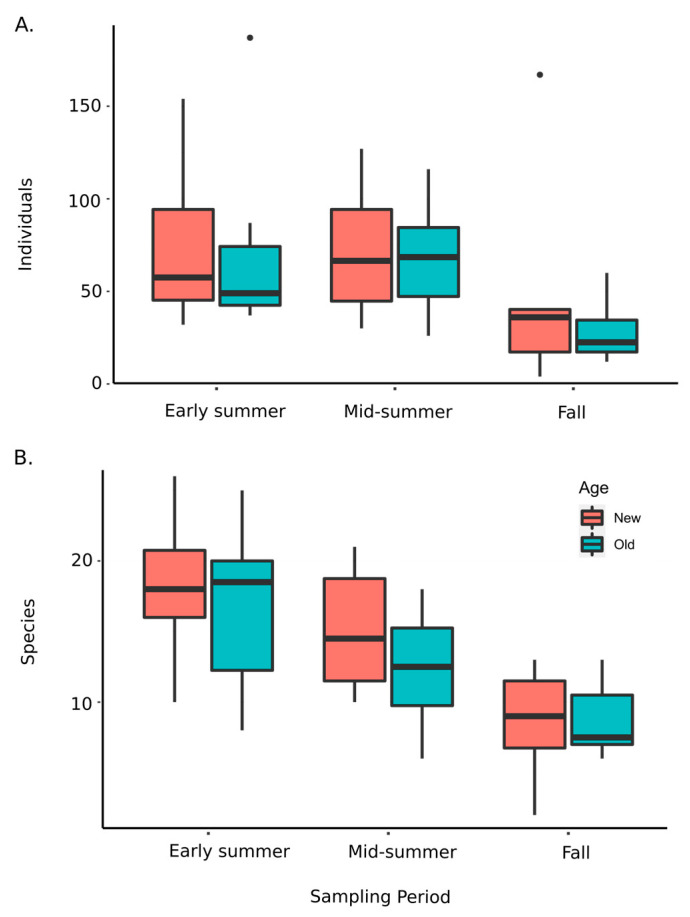
Box plots showing medians, quartiles, and ranges of beetle abundance (**A**) and richness (**B**) per site for new (“red”) and old (“blue”) woods over the three sampling periods. Outlier points are indicated by dots.

**Figure 7:**
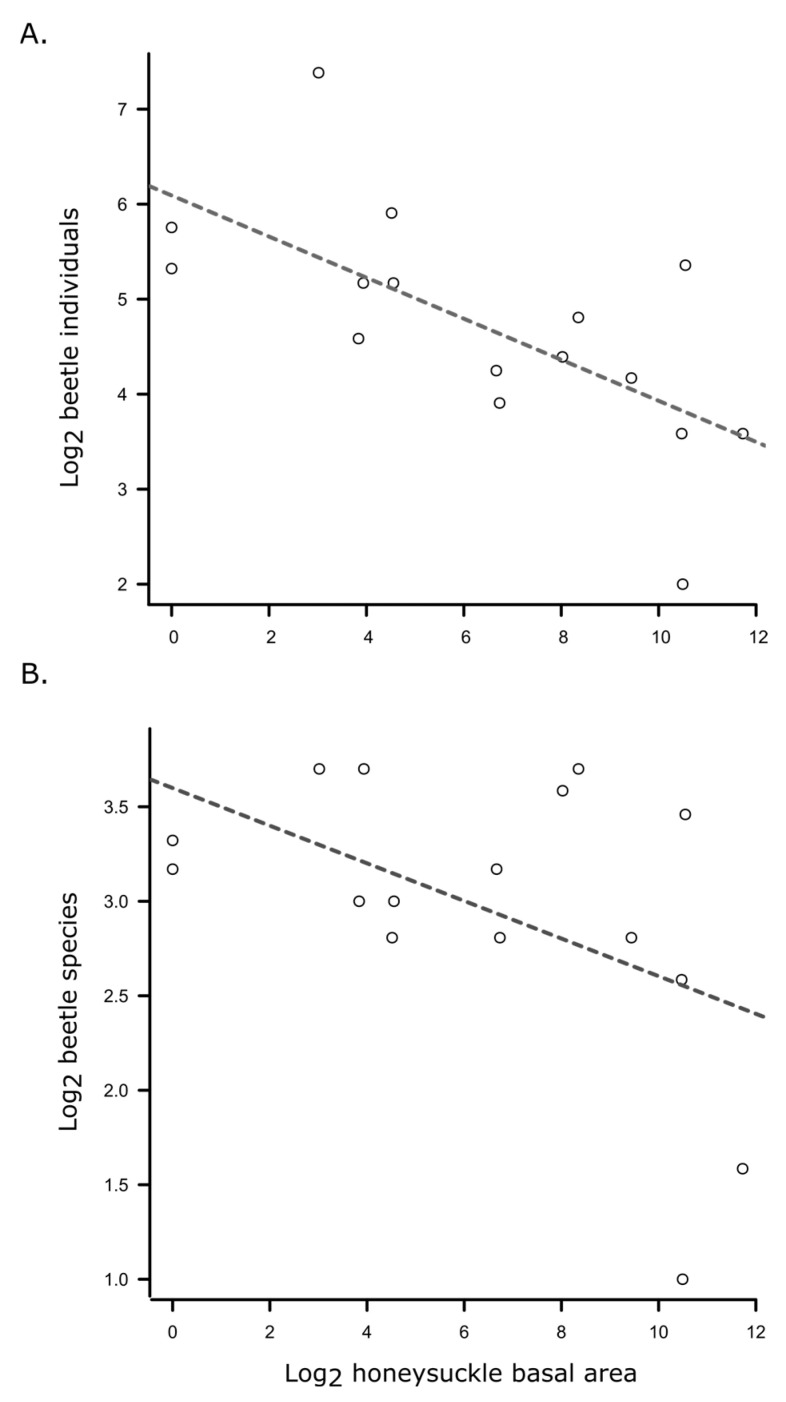
Linear regressions of beetle abundance (**A**) and richness (**B**) against invasive honeysuckle abundance (log_2_ (basal area + 1)) during the fall sampling period. Abundance: F_1,14_ = 11.01, *p* = 0.005, adj. R^2^ = 0.40; richness: F_1,14_ = 4.51, *p* = 0.053, adj. R^2^ = 0.19).

The optimal model to explain variation in beetle abundance included age, season, and honeysuckle as fixed effects, with park as a random effect. Beetle abundance significantly declined over the season (F_2,36_ = 13.074, *p* < 0.0001; Figure 6A) and with increasing honeysuckle basal area (F_1,38_ = 8.163, *p* = 0.007), but age did not have a statistically significant effect (F_1,38_ = 1.477, *p* = 0.232; Table 3). Although beetle abundance was generally lower with increasing honeysuckle invasion, the significance of this relationship was driven by the fall sampling period (Figure 7A). Coarse woody debris did not significantly affect beetle abundance and was not included in the final model. The extent of honeysuckle invasion varied widely among sampling sites (Table S1; F_7,64_ = 6.234, *p* < 0.001); however, overall, it was greater in young forest stands (when nested within site (F_8,64_ = 7.927, *p* < 0.001)).

The optimal model for beetle species richness included age, season, and honeysuckle as fixed effects, with park as a random effect. Beetle species richness declined over the season (F_2,36_ = 22.574, *p* < 0.0001; Table 3; Figure 6B) and with increasing honeysuckle (F_1,41_ = 8.622, *p* = 0.005). Again, this negative relationship with invasive honeysuckle was largely driven by the fall sampling period (Figure 7B). Richness tended to be higher in the young woods samples, but this was marginally not significant (F_1,37_ = 3.819, *p* = 0.058). Coarse woody debris had a stronger effect on richness than abundance. However, neither were significant, and the variable was excluded from the final model.

Consistent with previous studies [51], species richness was positively correlated with abundance (*p* < 0.0001) but with a relatively low adjusted R^2^ (0.38), and abundance accounted for less than half of the variation in richness. In contrast, the effective number of species showed almost no correlation with abundance (*p* = 0.839), as expected.

**Figure 8:**
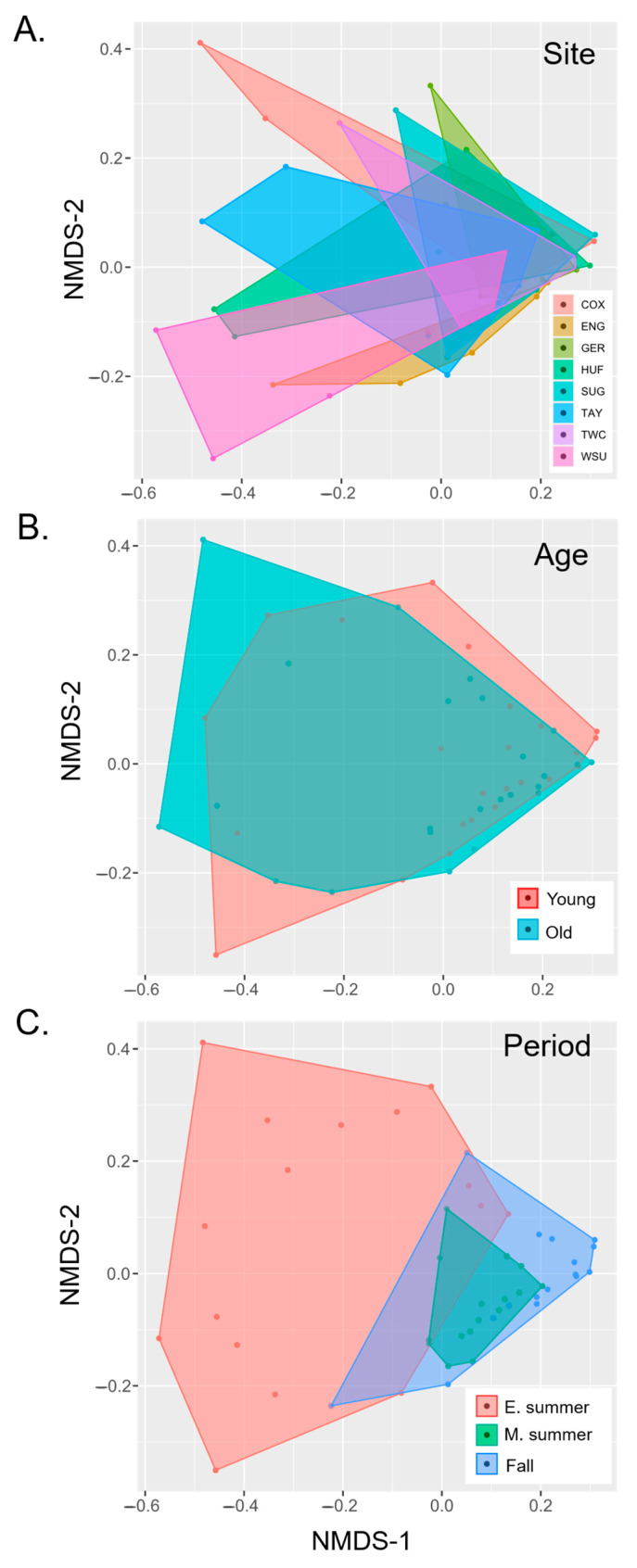
NMDS ordination for samples grouped by (**A**) site, (**B**) age class, and (**C**) sampling period (early summer, mid-summer, and fall). Colored dots represent samples, and shapes represent convex hulls relative to grouping factor.

Beetle species richness exhibited a marked, linear, seasonal decline over the sampling period (Figure 4D and Figure 6A). In contrast, abundance was similar over the first two sampling periods but dropped significantly in the fall sampling period (Figure 6B). NMDS ordination of samples revealed a weak but marginally significant effect of site on community composition (*R* = 0.066, *p* = 0.046; Figure 8A) but no evidence for an effect of forest age (R = −0.030; *p* = 0.987; Figure 8B). However, there was a clear and highly significant pattern of distinct communities between seasonal sampling periods with relatively strong separation between the early summer sampling period and the later sampling periods along NMDS axis 1 (*R* = 0.325, *p* < 0.001; Figure 8C). The overall Jaccard similarity between the old and young woods was 0.34. Of the 211 morphospecies, 83 were unique to the young woods, 57 were unique to the old woods, and 71 were shared. On average, Jaccard similarities between old and young woods within sites were the highest (0.23, S.D. = 0.08), followed by similarities between old woods among sites (0.20, S.D. = 0.05), young and old woods across sites (0.19, S.D. = 0.06), and young woods across sites (0.17, S.D. = 0.06; see Table S5).

Only 12 morphospecies (5.7%) were collected during all three sampling periods. The early and mid-summer sampling periods were most similar, sharing 40 morphospecies (22.4%), and the early summer and fall sampling periods were the least similar, sharing only 16 morphospecies (9.9%).

## 4. Discussion

### 4.1. Fungus-Associated Beetle Communities in Southwest Ohio Woodlands

The richness of beetles captured in this study, 211 morphospecies, is impressive given the small, specialized trap design and limited sampling durations. Still, this number of species represents less than half of the estimated 515 species that we might capture, suggesting that our study is just a beginning in documenting fungus-associated beetles in Southwest Ohio forests. As mentioned previously, we likely underestimated the total number of beetle species captured due to the presence of cryptic species, and we probably overestimated the number of species that were associated with fungi, as feeding preferences of beetles often vary widely, even within a family.

Over half of the recognized beetle morphospecies were singletons, which may give a false impression of the dissimilarity in beetle composition among sites. Greater sampling efforts may have recovered more specimens of these apparently rare species at other sites and sampling locations, and sampling over several seasons would provide more complete coverage and a better understanding of the phenology of the beetle community.

We expected to sample a greater abundance of species belonging to families with known direct, obligate fungal relationships, such as Cryptophagidae, Erotylidae, Mycetophagidae, and Tetratomidae. All of these families were present, yet in low numbers. We have personally collected Erotylidae frequently from oyster mushrooms. However, it is possible that the height of the trap from the ground (1 m) discouraged some taxa, or a greater volume of bait would be necessary to attract more of these taxa. Certain taxa of very small mycophagous beetles, such as Leiodidae, feed on subterranean fungi [52], and they may stay on or near the ground and be missed by sampling with hanging traps.

Interestingly, the beetle genus *Melanophthalma* (Latridiidae) was captured in by far the greatest abundance (1106), but it is not known to be associated with the fungal bait utilized in this study, *Pleurotus ostreatus*, the oyster mushroom [31]. This species typically feeds on fungal spores from various families of fungi and is typically collected from leaf litter. *Melanophthalma* may have been attracted to the odor of oyster mushrooms because these fungi are associated with decaying wood, which may be a suitable substrate of their preferred fungi as well. Notably, a closely related genus of Latridiidae, *Corticarina*, has been recorded in association with oyster mushrooms [31]. Although the family Latridiidae was the most abundant, there were only four recognized morphospecies in our samples. This may be an accurate assessment; however, a closer examination by a latridiid expert might reveal more species that are superficially similar in morphology.

### 4.2. Effects of Forest Variables

We found no support for our hypothesis that older forest stands possess greater diversity and abundance of mycophilous beetles. In contrast, there was a nearly significant trend in the opposite direction with younger forests hosting a greater species richness of beetles. Perhaps this trend could be attributable to greater diversity of resources in younger woods harboring both early successional and late successional tree species and a peak in fungal diversity early in succession (e.g., [53]). It is possible that mycophilous beetle richness in forests does increase with forest age, however, the relationship is asymptotic, and our young forest sites were too old (or too similar in age to the older forest sites) to detect this relationship. Furthermore, a more accurate assessment of mean and maximum tree ages using increment borers might allow finer resolution of the effect of forest age as a continuous instead of categorical variable. A survey of polyporoid and corticioid fungi in the northern Midwest U.S. found no significant difference in fungal richness relative to forest management history (old growth, selectively logged, or previously clearcut [65–70 years]) [8], and meta-analysis suggests that ectomycorrhizal fungi communities tend to recover to old-growth richness levels within about 90 years after forest disturbance (and in many cases far more quickly) [54]. This meta-analysis further indicates recovery of saproxylic beetle communities (which tend to be fungal associated) within about 60 years [54]. Woodlands in this heavily developed region of Southwest Ohio are highly fragmented. Preliminary regression analyses found no indication that contiguous forest area of our sites influenced mycophilous beetle abundance and richness (*p* > 0.13), yet it is possible that the effects of this fragmentation overshadow variation between mature and young forest stands within these forest patches.

We expected to find a greater volume of coarse woody debris (CWD) in the older forests, which has been shown to have a positive effect on the richness of saproxylic organisms [34]; however, we found no clear differences in amounts of CWD between the younger and older forest stands. Neither did we find any significant effects of CWD on abundance or richness of fungus-associated beetles. It is possible that even our young forest stands were sufficiently old that CWD was not a limiting resource for fungi and their associated beetles or that some other unmeasured factor was more limiting. It is also possible that our measures of CWD were too limited (focused around our traps), and that more extensive surveys of the sampling sites would be needed to accurately assess available CWD levels.

Season (sampling period) was the most important variable in determining beetle community composition, consistent with previous studies of Coleoptera [55] and Lepidoptera [7]. The strongest separation in community composition is between the early summer fauna and the later summer and fall sampling periods, which appeared nested (Figure 8C). In the final sampling period, during the first week of October, beetle abundance and richness was notably depressed. This may have been due to low precipitation in the weeks previous to the sampling period, which is known to affect the abundance of fungal sporocarps [56,57].

We found that both abundance and richness of mycophilous beetles declined with the increasing incidence of the invasive shrub Amur honeysuckle (*L. maackii*). The abundance of honeysuckle was highly variable among sites in both young and old forest stands, and the effects on beetle communities were only clearly discernable in the fall sampling period (although negative trends were observed across sampling periods). It is not clear if this is a causative relationship or a correlative relationship associated with other aspects of the forest stands, but honeysuckle has been shown to negatively affect both native vascular plant communities [58] and the abundance of mycorrhizal fungi [55,56,57,58,59]. Interestingly, levels of honeysuckle infestation were, on average, greater in younger forest stands, but the effects of honeysuckle were independent of stand age. The observation that the negative relationship was only clearly discernable in fall suggests that honeysuckle may exacerbate negative effects of drought on mycophilous beetles, or it may be that underlying negative effects are masked by other factors during the summer growing season. Discerning the nature of the effects of honeysuckle on the fungus-associated beetle community would require a carefully controlled longitudinal study over several years, as the effects of adding or removing honeysuckle are not immediately manifested. Honeysuckle has been shown to discourage seedlings of native trees [37], which would alter the forest composition as older trees die without replacement. In the long term, this would reduce the suitable habitat for many fungus-associated beetles. It may also be that the elevated abundance of honeysuckle is associated with another existing variable (e.g., disturbance) that is itself unfavorable to mycophilous beetles.

Much opportunity remains to expand our knowledge of mycophilous beetle communities in Southwest Ohio and other forests of Eastern North America and their relationship to forest characteristics. Using the same type of traps and bait over a full year instead of only three discrete trapping events would capture more species and, perhaps more interestingly, allow more nuanced examination of changes in seasonal abundance of various taxa. A different species of bait fungus could also be used, which might attract a different set of beetles. Baited pitfall traps would also capture a different assemblage of beetles, as some stay close to the forest floor and feed on subterranean fungal sporocarps. Finer-scale identification of beetle taxa and a more nuanced approach to their inclusion as “mycophilous” might also provide better resolution of community patterns in relation to forest traits. Perhaps one of the best ways would be active sampling of sporocarps. This would be labor intensive and difficult to standardize across sites but has the benefits of direct beetle–fungal host association and very little bycatch.

## 5. Conclusions

There are many challenges to preserving the diversity and ecosystem services forest detritivore food webs. Documenting what taxa are present and how communities vary with forest characteristics represent the initial steps required to preserve such biodiversity. Our study of mycophilous Coleoptera is one such step. Even though we found no clear relationship between forest age and abundance or diversity of mycophilous beetles, our results indicate that woody plant invasion is associated with lower beetle richness and suggest that their taxonomic composition may differ between old and young woods. Preserving the remaining contiguous areas of older growth forests and maintaining a diversity of successional stages in other areas will likely help to maintain biodiversity in the region. The removal of invasive honeysuckle will likely benefit the beetle community as well as many other groups of organisms. The diversity of mycophilous beetles is directly tied to the success of their fungal hosts, which can be affected by many environmental variables that need to be explored in more detail. Some beetles associated with fungi may have to adapt to changing climate as well, as there is support for warmer temperatures altering fungal fruiting times [60].

## Figures and Tables

**Table 1:** Richness and abundance of Coleoptera by site. Richness totals do not sum, as there are shared morphospecies.

Metric	Age Class	COX	ENG	GER	HUF	SUG	TAY	TWC	WSU	Total
Richness	Young	39	22	45	47	35	42	30	23	154
	Old	43	36	22	34	25	36	19	38	128
	**Total**	**61**	**50**	**55**	**64**	**52**	**60**	**41**	**53**	**211**
Abundance	Young	282	74	302	307	119	147	169	124	1524
	Old	231	172	140	315	106	128	130	127	1349
	**Total**	**513**	**246**	**442**	**622**	**225**	**275**	**299**	**251**	**2873**

**Table 2:** Estimated average age and average maximum age of trees in years at each site. Sites where the average tree age for the older forest was not greater than the younger are indicated with (*).

Site	Age Class	Average Max. Age	Average Age
COX	Old	109	63
	Young	69	47
ENG	Old	98 *	58 *
	Young	126	65
GER	Old	91	81
	Young	80	55
HUF	Old	171	92
	Young	140	57
SUG	Old	104	47 *
	Young	71	66
TAY	Old	122	69
	Young	76	48
TWC	Old	114	75
	Young	100	64
WSU	Old	118	80
	Young	73	52

**Table 3:** Results of linear mixed models for abundance and richness. New age and sampling period 1 are base intercepts.

Abundance	Estimate	df	t Value	Pr(>|t|)
Age	−0.2951	38	−1.215	0.2318
Mid-summer	0.0200	36	0.070	0.9442
Fall	−1.2547	36	−4.393	*<0.0001*
Honeysuckle	−0.0004	38	−2.857	*0.0069*
Log(CWD) *	0.0619	39	0.391	0.6978
**Richness**				
Age	−2.2102	38	−1.954	*0.0582*
Mid-summer	−3.7500	36	−2.828	*0.0076*
Fall	−8.8750	36	−6.692	*<0.0001*
Honeysuckle	−0.0021	41	−2.936	*0.0054*
Log(CWD)*	0.8196	39	1.099	0.2787

* CWD was not included in optimal model, but it is listed here for information purposes.

## Data Availability

The beetle survey and site characteristic data presented in this study are openly available in Dryad at DOI: 10.5061/dryad.vq83bk45j.

## Supplementary Materials

The following supporting information can be downloaded at: https://www.mdpi.com/article/10.3390/insects16070735/s1. Table S1: GPS coordinates for study sites and locations. Table S2: Growth factors for tree species at our study sites. Table S3: List of beetle species/morphospecies recovered by family. Table S4: Observed and estimated species richness and diversity indices by age and sampling period. Table S5: Jaccard similarity indices for all sites. Figure S1: Decay class and volume in cm^3^ of coarse woody debris.
