# Supplementary material for: Effects of Forest Age and Invasive Shrubs on Mycophilous Coleoptera Communities in a Temperate Deciduous Woodland
Source: Insects. 2025 Jul 18;16(7):735. doi: 10.3390/insects16070735 (PMC12296189; doi:10.3390/insects16070735)

Supplementary Materials

Supplementary Table S1: GPS Coordinates for study sites and locations, approximate forest stand size (approximate area of contiguous woodland in which both young and old survey plots were located), summed basal area (cm^2) of invasive honeysuckle for plots, and tree species present in plots that were measured for DBH.

**Site**	**Stand size (ha)**	**Age class**	**Latitude**	**Longitude**	**Honeysuckle**	**Tree species measured**
Cox Arboretum	50	Young	39.6542	-84.229	14.3	*Acer saccharum, Quercus alba, Q. montana, Ulmus americana, Carya ovata, Juniperus virginiana*
		Old	39.6579	-84.2319	325.6	*A. saccharum, Q. alba, U. americana*
Englewood	550	Young	39.8809	-84.2825	3391.9	*A. saccharum, Maclura pomifera, Robinia pseudoacacia*
		Old	39.8876	-84.2854	21.9	*A. saccharum, Tilia americana*
Germantown	820	Young	39.6355	-84.4081	7.1	*A. saccharum, Q. rubra*
		Old	39.6410	-84.4218	13.3	*A. saccharum, Fagus grandifolia*
Huffman	180	Young	39.7995	-84.0908	0	*A. saccharum, Populus deltoides, Aesculus glabra*
		Old	39.7998	-84.0873	1421.2	*A. saccharum, P. deltoides, Juglans nigra, Gleditsia triacanthos*
Sugarcreek	200	Young	39.6184	-84.0979	1497.3	*A. saccharum, Prunus serotina*
		Old	39.6239	-84.0958	693.6	*A. saccharum, Celtis occidentalis, Q. rubra, Fa. grandifolia,*
Taylorsville	310	Young	39.8733	-84.1614	100.6	*A. saccharum, Fa. grandifolia, U. americana, Fraxinus quadrangulata*
		Old	39.8872	-84.1558	105.5	*A. saccharum, Fa. grandifolia, Q. rubra, C. ovata*
Twin Creek	380	Young	39.5743	-84.3528	22.5	*A. saccharum, P. serotina, Fr. quadrangulata, C. cordiformis*
		Old	39.5943	-84.3537	0	*A. saccharum, Q. alba, aF. grandifolia, P. serotina*
WSU	95	Young	39.7861	-84.0521	1440.7	*A. saccharum, P. serotina*
		Old	39.7803	-84.0560	259.5	*A. saccharum, Q. muehlenbergii, Jug. nigra*

Supplementary Table S2: Growth factors for tree species at our study sites. Growth Factor x DBH in inches gives a rough estimate of tree age

Tree Species	Growth Factor
American Basswood	3
American Beech	6
American Elm	4
Bitternut Hickory	7.5
Black Cherry	5
Black Locust	3
Black Walnut	4.5
Blue Ash	5
Chestnut Oak	5.5
Chinkapin oak	6
Cottonwood	2
Eastern Red Cedar	4
Hackberry	3.5
Honey Locust	3
Northern Red Oak	4
Ohio Buckeye	5
Osage Orange	4
Shagbark Hickory	7.5
Sugar Maple	5.5
Sycamore	4
White Oak	5

Supplementary Table S3. List of beetle species sampled and morphospecies by family. Names highlighted in green are morphospecies designations, those in names in italics without highlighting were identified to genus or species level. Within a family, "Unknown genus" may represent multiple genera.

Family	Genus	Species	Quantity
**Anthicidae**			
	Anthicus	*cervinus* LaFerté-Sénectère, 1849	1
	Notoxus	sp. 1 (NotoOne)	4
**Buprestidae**			
	Agrilus	sp. 1 MedGold	2
	Agrilus	sp. 2 NotEAB	1
	Agrilus	sp. 3 ProKeel	3
	Agrilus	sp. 4 Small DarkGreen	7
**Ciidae**			
	Unknown genus	sp. 1 SmallHorns	1
**Cleridae**			
	Cregya	*mixta* (LeConte), 1866	1
	Cymatodera	*bicolor* (Say), 1825	1
	Enoclerus	*nigripes* (Say), 1823	1
	Madoniella	*dislocata* (Say), 1825	115
	Pyticeroides	*laticornis* (Say), 1835	188
**Coccinellidae**			
	Brachiacantha	*quadripunctata* Melsheimer, 1847	1
	Microweisea	sp. 1 TinyTiny	2
	Scymnus	sp. 1 HairyRound	1
**Corylophidae**			
	Holopsis	sp. 1 ClearFront	2
	Orthoperus	sp. 1 TooSmall	3
	Sericoderus	*lateralis* (Gyllenhal), 1827	1
	Unknown genus	sp. 1 Small Hooded	1
**Cryptophagidae**			
	Cryptophagus	sp. 1 ProCircle	3
**Curculionidae**			
	Conotrachelus	*anaglypticus* (Say), 1831	1
	Cyrtepistomus	*castaneus* (Roelofs), 1873	1
	Dryophthorus	*americanus* Bedel, 1885	7
	Pseudoedophrys	*hilleri* (Faust), 1889	2
	Phloeotribus	sp. 1 PseudoClub	3
	Scolytus	sp. 1 Scoly FlatHead	6
	Stenoscelis	*brevis* (Boheman), 1845	4
	Xyleborus	sp. 1 Scoly One	486
	Unknown genus	sp. 2 Bigeye tucknose	12
	Unknown genus	sp. 3 HairySmall	4
	Unknown genus	sp. 4 Notch Tucknose	4
	Unknown genus	sp. 5 Scoly FlatClub	2
	Unknown genus	sp. 6 Scoly Medium	4
	Unknown genus	sp. 7 Scoly Skinny	36
	Unknown genus	sp. 8 Scoly Tiny	30
	Unknown genus	sp. 9 ScolyLong	1

	Unknown genus	sp. 10 Smalleye Tucknose	2
	Unknown genus	sp. 11 WhiteCenter	1
**Elateridae**			
	Ampedus	*areolatus* (Say), 1823	3
	Ampedus	sp. 1 HairyBall	3
	Ampedus	*nigricollis* (Herbst), 1801	1
	Ampedus	*semicinctus* (Randall), 1838	1
	Athous	sp. 1 LongNotum	8
	Glyphonyx	sp. 1 CurveAngle	7
	Hemicrepidius	sp. 1 BigBoy	1
	Horistonotus	*curiatus* (Say), 1839	1
	Idolus	sp. 1 Nondescript	3
	Lacon	*discoideus* (Weber), 1801	1
	Melanotus	sp. 1 BlackLong	1
	Melanotus	sp. 2 BrownChopped	1
	Unknown genus	sp. 1 Constricted	3
	Unknown genus	sp. 2 Edges	1
	Unknown genus	sp. 3 GoldStraight	1
	Unknown genus	sp. 4 HairyPaleEdge	1
	Unknown genus	sp. 5 LongHair	1
	Unknown genus	sp. 6 Straight	2
**Erotylidae**			
	Acropteroxys	*gracilis* (Newman), 1838	1
	Dacne	*quadrimaculata* (Say), 1835	1
	Glischrochilus	*sanguinolentus* (Olivier), 1790	1
	Toramus	*pulchellus* (LeConte), 1866	1
	Triplax	*festiva* Lacordaire, 1842	1
	Triplax	*flavicollis* Lacordaire, 1842	19
	Triplax	*thoracica* Say, 1825	3
	Tritoma	*sanguinipennis* (Say), 1825	1
**Eucnemidae**			
	Isorhipis	*obliqua* (Say), 1839	45
	Unknown genus	sp. 1 Alternate	1
	Unknown genus	sp. 2 BrownBlack	1
	Unknown genus	sp. 3 FlatBlack	2
	Unknown genus	sp. 4 GoldenHair	1
	Unknown genus	sp. 5 LittleSquare	3
	Unknown genus	sp. 6 Serrate	1
	Unknown genus	sp. 7 SparseRound	1
	Unknown genus	sp. 8 Tube BlackBack	18
	Unknown genus	sp. 9 TwoLine	3
**Histeridae**			
	Teretrius	sp. 1 Lollipop	1
	Unknown genus	sp. 1 Clown3.5	15
	Unknown genus	sp. 2 Digger	3
**Laemophloeidae**			
	Charaphloeus	sp. 1 *TeaCup*	11
	Laemophloeus	*biguttatus* (Say), 1825	1
	Laemophloeus	*megacephalus* Grouvelle, 1876	1
	Laemophloeus	sp. 1 StrongLine	1

	Unknown genus	sp. 1 Laemo One	16
**Latridiidae**			
	Corticaria	sp. 1 *ToothNeck*	1
	Melanophthalma	sp. 1 *Little Brown*	1105
	Melanophthalma	sp. 2 *Little DarkBrown*	1
	Stephostethus	sp. 1 *Hourglass*	5
**Leiodidae**			
	Leiodes	sp. 1 *Solo*	1
	Unknown genus	sp. 1 Smashed	1
**Lycidae**			
	Calopteron	*terminale* (Say), 1823	1
**Melandryidae**			
	Dircaea	*liturata* LeConte, 1866	3
**Monotomidae**			
	Bactridium	sp. 1 ShinyLine	6
	Europs	*pallipennis* (LeConte), 1861	2
	Rhizophagus	sp. 1 SquareNotum	4
**Mordellidae**			
	Mordellaria	*serval* (Say), 1835	13
	Mordellaria	*undulata* (Melsheimer), 1845	6
	Mordellistena	*limbalis* (Melsheimer), 1845	2
	Mordellistena	sp. 1 NoPattern	19
	Yakuhananomia	*bidentata* (Say), 1824	2
	Unknown genus	sp. 1 Batman	2
	Unknown genus	sp. 2 Black	7
	Unknown genus	sp. 3 FadeToBlack	128
	Unknown genus	sp. 4 Heart	3
	Unknown genus	sp. 5 JetFighter	17
	Unknown genus	sp. 6 Monster	1
	Unknown genus	sp. 7 OrangeBlackSpot	6
	Unknown genus	sp. 8 OrangeShoulder	11
	Unknown genus	sp. 9 Pale	8
	Unknown genus	sp. 10 RedStripe	1
	Unknown genus	sp. 11 SixStripe	1
	Unknown genus	sp. 12 StripeBareLeg	8
	Unknown genus	sp. 13 Tiger	4
	Unknown genus	sp. 14 Triangle	3
	Unknown genus	sp. 15 TwoTone	8
	Unknown genus	sp. 16 VelvetSpots	1
	Unknown genus	sp. 17 YellowShoulder BlackSpot	1
**Mycetophagidae**			
	Litargus	sp. 1 BrownMottled	15
	Litargus	sp. 2 Shadow	1
	Litargus	*tetraspilotus* LeConte, 1856	4
	Mycetophagus	*punctatus* Say, 1826	2
	Mycetophagus	sp. 1 SandySpot	1
	Mycetophagus	*serrulatus* Casey, 1900	1
	Unknown genus	sp. 1 Yellow	1
**Nitidulidae**			
	Amphicrossus	*ciliates* (Olivier), 1811	2

	Carpophilus	*antiquus* Melsheimer, 1844	7
	Carpophilus	sp. 1 BlackBrownEdge	13
	Carpophilus	sp. 2 OldBrown	11
	Colopterus	*semitectus* (Say), 1825	3
	Colopterus	*unicolor* (Say), 1825	1
	Epuraea	sp. 1 BrownSmooth	2
	Glischrochilus	*fasciatus* (Olivier), 1790	27
	Glischrochilus	*quadrisignatus* (Say), 1835	1
	Glischrochilus	*sanguinolentus* (Olivier), 1790	13
	Omosita	*nearctica* Kirejtshuk, 1987	1
	Prometopia	*sexmaculata* (Say), 1825	2
	Stelidota	*geminate* (Say), 1825	15
	Unknown genus	sp. 1 Light Brown	1
	Unknown genus	sp. 2 FlatTan	1
**Phalacridae**			
	Olibrus	sp. 1 RoundBrown	5
	Stilbus	sp. 2 Shiny	2
**Ptilodactylidae**			
	Ptilodactyla	sp. 1 BrownFade	6
**Ptinidae**			
	Caenocara	*oculatum* (Say), 1824	1
	Trichodesma	*klagesi* Fall, 1905	1
	Unknown genus	sp. 1 Antlers	2
	Unknown genus	sp. 2 FakeScarab	1
	Unknown genus	sp. 3 GoldFur	1
	Unknown genus	sp. 4 SnaggleTooth	1
**Pyrochroidae**			
	Dendroides	*canadensis* Latreille, 1810	1
	Neopyrochroa	*flabellate* (Fabricius), 1787	5
**Scarabaeidae**			
	Aphodius	sp. 1 ShovelNose	1
**Silphidae**			
	Nicrophorus	*orbicollis* Say, 1825	1
**Silvanidae**			
	Silvanus	*muticus* Sharp, 1899	15
**Staphylinidae**			
	Bisnius	sp. 1 LongBBOO	66
	Bisnius	sp. 2 LongBOBO	11
	Carphacis	*dimidiatus* (Erichson), 1839	1
	Euconnus	sp. 1 Euconnus One	2
	Hesperus	*apicialis* (Say), 1830	4
	Lordithon	sp. 1 Lordithon Vague	1
	Lordithon	sp. 2 Dark	1
	Palaminus	sp. 1 SuperCool	1
	Philonthus	*caeruleipennis* (Mannerheim), 1830	5
	Pycnoglypta	*campbelli* Gusarov, 1995	11
	Sepedophilus	sp. 1 GoldComb	3
	Sepedophilus	sp. 2 Nice5mm	1
	Sepedophilus	sp. 3 SmallComb	1
	Sepedophilus	sp. 4 Sepedophilus	1

	Siagonium	*americanum* (Melsheimer), 1844	1
	Upoluna	*batrisioides* (Motschulsky), 1856	1
	Unknown genus	sp. 1 BHeadFur	6
	Unknown genus	sp. 2BNNN	11
	Unknown genus	sp. 3 GemStone	1
	Unknown genus	sp. 4 SmallLong	4
**Synchroidae**			
	Synchroa	*punctata* Newman, 1838	1
**Tenebrionidae**			
	Anaedus	*brunneus* (Ziegler), 1844	1
	Meracantha	*contracta* Beauvois, 1813	1
	Unknown genus	sp. 1 CombClaw One	1
**Tetratomidae**			
	Hallomenus	*scapularis* Melsheimer, 1846	1
	Penthe	*pimelia* (Fabricius), 1801	1
**Throscidae**			
	Aulonothroscus	*pugnax* (Horn), 1885	2
**Trogossitidae**			
	Unknown genus	sp. 1 Shovelnose	4
**Zopheridae**			
	Synchita	*parvula* Guérin-Méneville, 1844	1

Supplementary Table S4. Observed and estimated species richness and diversity indices by age and sampling period, Shannon diversity is calculated as effective species e^{H}.

Subset	Diversity	Observed	Estimated	S.E.	LCL	UCL
Total	Species richness	211	515	89.3	384	745
	Shannon diversity	16.8	18.3	0.7	16.8	19.7
	Simpson diversity	5.4	5.4	0.2	5.4	5.8
Young forest	Species richness	154	318	51.5	244	453
	Shannon diversity	15.4	17.1	0.9	15.4	18.9
	Simpson diversity	5.0	5.0	0.2	5.0	5.4
Old forest	Species richness	128	260	47.9	194	391
	Shannon diversity	16.1	17.7	1.0	16. 1	19.7
	Simpson diversity	5.8	5.8	0.3	5.8	6.3
Early summer sample period	Species richness	124	328	77.8	223	544
	Shannon diversity	17.3	19.6	1.1	17.4	21.8
	Simpson diversity	7.5	7.6	0.3	7.5	8.2
Mid-summer sample period	Species richness	95	269	75.531	172	488
	Shannon diversity	9.4	10.5	0.7	9.4	11.9
	Simpson diversity	3.8	3.8	0.2	3.8	4.1
Fall sample period	Species richness	53	174	84.9	88	471
	Shannon diversity	6.5	7.3	0.7	6.5	8.7
	Simpson diversity	2.5	2.5	0.2	2.5	2.8

Supplementary Table S5. Jaccard similarity indices for all sites. The shaded cells are a comparison between young and old of the same park. Values above represent comparison of young (row) to old (column). Values below represent comparison of young to young.

	COX	ENG	GER	HUF	SUG	TAY	TWC	WSU
**COX**	0.34	0.19	0.17	0.26	0.21	0.23	0.16	0.28
**ENG**	0.09	0.16	0.19	0.10	0.12	0.16	0.17	0.07
**GER**	0.24	0.14	0.22	0.22	0.19	0.25	0.14	0.26
**HUF**	0.26	0.10	0.28	0.27	0.20	0.22	0.12	0.23
**SUG**	0.23	0.12	0.16	0.15	0.15	0.15	0.13	0.20
**TAY**	0.21	0.12	0.23	0.25	0.13	0.32	0.13	0.29
**TWC**	0.19	0.11	0.15	0.15	0.18	0.14	0.20	0.17
**WSU**	0.17	0.15	0.13	0.15	0.07	0.23	0.10	0.15

Supplementary Figures

Supplementary Figure S1: Decay class and volume in cm^3 of coarse woody debris

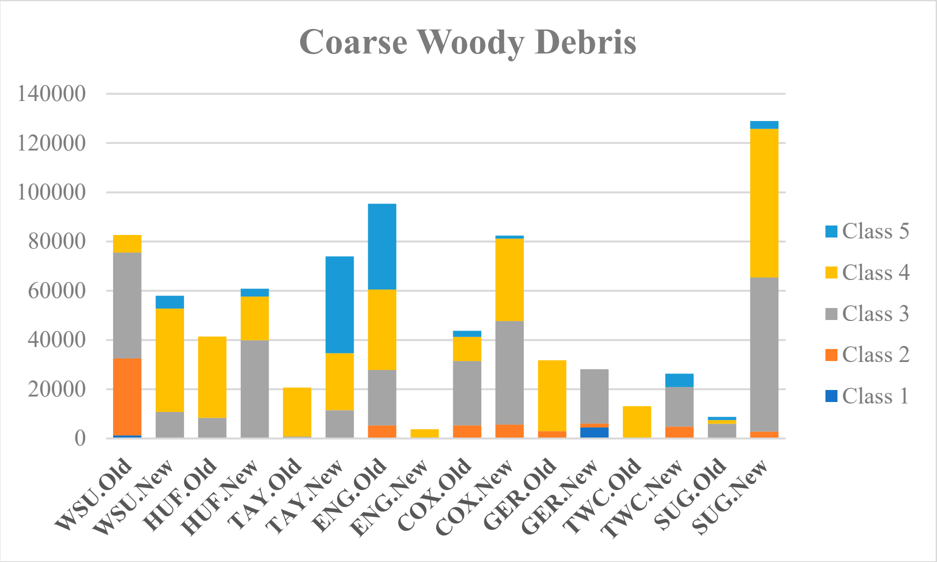

Supplement: Supplementary file 1 [file insects-16-00735-s001.zip › insects-3597163-supplementary.pdf]
